# Prognostic value of genomic alterations in minimal residual cancer cells purified from the blood of breast cancer patients

**DOI:** 10.1054/bjoc.2000.1501

**Published:** 2000-12

**Authors:** F Austrup, P Uciechowski, C Eder, B Böckmann, B Suchy, G Driesel, S Jäckel, I Kusiak, H-J Grill, M Giesing

**Affiliations:** Institut für Molekulare NanoTechnologie, Berghäuser Str. 295, Recklinghausen, 45659, Germany

**Keywords:** minimal residual cancer cells, blood, genomic imbalances, breast cancer prognosis

## Abstract

The prognostic value of disseminated tumour cells derived from 353 breast cancer patients was evaluated. Disseminated tumour cells were purified from blood using a newly established method and nucleic acids were subsequently isolated. We investigated genomic imbalances (GI) such as mutation, amplification and loss of heterozygosity of 13 tumour suppressor genes and 2 proto-oncogenes using DNA from isolated minimal residual cancer cells. Significant correlations were found between genomic alterations of the *DCC* - and *c-erbB-2* genes in disseminated breast cancer cells and actuarial relapse-free survival. Furthermore, increasing numbers of genomic imbalances measured in disseminated tumour cells were significantly associated with worse prognosis of recurrent disease. Logistic regression and Cox multivariate analysis led to the identification of genomic imbalances as an independent prognostic factor. Determination of disseminated tumour cells by genotyping of oncogenes and tumour suppressor genes seems not only to be a useful adjunct in follow up of carcinoma patients but provides also valuable additional individualized prognostic and predictive information in breast cancer patients beyond the TNM system. © 2000 Cancer Research Campaign http://www.bjcancer.com


*British Journal of Cancer* (2000) **83**(12), 1664–1673
© 2000 Cancer Research Campaign
doi: 10.1054/ bjoc.2000.1501, available online at http://www.idealibrary.com on IDEAL®

http://www.bjcancer.com

### Prognostic value of genomic alterations in minimal residual cancer cells purified from the blood of breast cancer patients

**F Austrup, P Uciechowski, C Eder, B Böckmann, B Suchy, G Driesel, S Jäckel, I Kusiak, H-J Grill and M Giesing**

Institut für Molekulare NanoTechnologie, Berghäuser Str. 295, 45659 Recklinghausen, Germany

**Summary** The prognostic value of disseminated tumour cells derived from 353 breast cancer patients was evaluated. Disseminated tumour cells were purified from blood using a newly established method and nucleic acids were subsequently isolated. We investigated genomic imbalances (GI) such as mutation, amplification and loss of heterozygosity of 13 tumour suppressor genes and 2 proto-oncogenes using DNA from isolated minimal residual cancer cells. Significant correlations were found between genomic alterations of the *DCC*- and *c-erbB-2* genes in disseminated breast cancer cells and actuarial relapse-free survival. Furthermore, increasing numbers of genomic imbalances measured in disseminated tumour cells were significantly associated with worse prognosis of recurrent disease. Logistic regression and Cox multivariate analysis led to the identification of genomic imbalances as an independent prognostic factor. Determination of disseminated tumour cells by genotyping of oncogenes and tumour suppressor genes seems not only to be a useful adjunct in follow up of carcinoma patients but provides also valuable additional individualized prognostic and predictive information in breast cancer patients beyond the TNM system. © 2000 Cancer Research Campaign http://www.bjcancer.com

**Keywords:** minimal residual cancer cells; blood; genomic imbalances; breast cancer prognosis

Breast cancer is by far the most common cancer in women. Despite progress in surgical techniques and treatment, many patients will relapse (Nemoto et al, 1980). For patients without evidence of systemic dissemination after primary surgery, prognostic parameters such as tumour size, nodal status, grading, hormonal receptor expression and others are determined. Using these parameters a statistical assessment of the risk of relapse leads to the decision whether to give systemic adjuvant chemotherapy or not. Undetected disseminated cancer cells can contribute to the failure of primary treatment. Moreover, the success of different therapeutic regimes is limited, as even cancer therapy is not always able to completely eradicate all neoplastic cells disseminated from the primary tumour. Therefore, detection of remaining minimal residual disease in individual patients could have an important clinical impact on assessment of prognosis and therapy outcome for breast cancer patients.

Genesis of a sporadic tumour is a multistage process involving activation of oncogenes and inactivation of tumour-suppressor genes (Lizard Nacol et al, 1997). Some tumour cells invade and generate metastases via either lymphatic or blood vessels. After primary resection surgery minimal residual cancer cells (MRCC) are the actual targets for chemotherapeutic drugs. Until now several attempts have been made to detect or isolate disseminated tumour cells from peripheral blood or bone marrow (Nawroz et al, 1996; Denis et al, 1997; Brandt et al, 1998; Leitzel et al, 1998; Naume et al, 1998; Soria et al, 1999). In nearly all analytical approaches, RT-PCR analyses were used to detect micrometastatic, disseminated cancer cells. Contrasting to the analysis of RNA, determination of genomic imbalances (GI) using DNA needs extremely pure cancer cells. Therefore, we established a new density and size-dependent method to isolate MRCC from blood. The method is based on observations that extravasation of tumour cells often occurs in clusters of malignant cells and clotting in the blood appears by aggregation of tumour cells and thrombocytes leading to the formation of large particles (Friedl et al, 1995; Aigner et al, 1997; Brandt et al, 1998). Furthermore, aggregated tumour cells in circulation may be a result of released, formally endothelial-attached micrometastases (Al-Mehdi et al, 2000). Identification and characterization of purified disseminated tumour cells in our approach is based on the analysis of genomic DNA. By this we exclude any problems of RT-PCR assays resulting from basal transcription of so called ‘tumour-specific’ and ‘tumour-associated’ genes of normal cells avoiding false positive findings (Lopez Guerrero et al, 1997; Zippelius et al, 1997). To determine tumour cells we measured genomic imbalances (GI) of 15 different genes; 13 tumour suppressor genes and 2 oncogenes. Once a tumour suppressor gene is inactivated on one allele loss of heterozygosity may uncover a phenotypic recessive mutational event in the remaining allele (Heide et al, 1997), a process which often occurs in early tumorigenesis. In the present study, we applied PCR-based detection of GI in archived DNA derived from isolated MRCC of 353 breast cancer patients. The prognostic relevance of MRCC with respect to the number and kind of genomic imbalances was subsequently evaluated.

### MATERIALS AND METHODS

### Patients and tumour characteristics

Since 1993 we have analysed disseminated tumour cells in blood and bone marrow of mainly breast and colon cancer patients. After

*Received 16 November 1999*
*Revised 14 August 2000*
*Accepted 17 August 2000*

*Correspondence to: M Giesing*

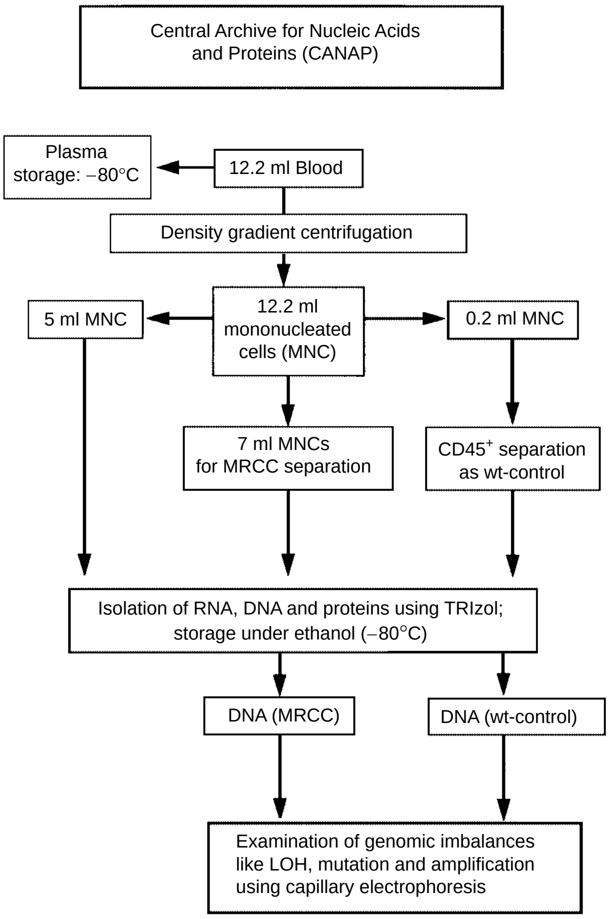

**Figure 1** Schematic diagram of blood fractionation and storage in the central archive of nucleic acids and proteins (CANAP) as described in Materials and methods diagnosis, surgery and primary care of the patients in University hospitals and specialized oncological hospitals, the patients follow-up was done by specialized oncologists. Blood samples were provided by several oncological centres of excellency in an open-cohort study. Detailed patients data (diagnosis, histology, treatment regiments, clinical follow-up data) were provided by a questionnaire.

For long-term storage of patients samples, we established a central archive for nucleic acids and proteins (CANAP) (Figure 1). This archive contains serum samples, purified RNA, DNA and proteins from isolated CD45 positive lymphocytes, MNCs and separated MRCC from patients with different malignant disorders. Using this archive as well as a yearly updated clinical database, we are able to analyse different parameters of interest by e.g. PCR analyses.

In the following investigation, DNA-samples stored in CANAP extracted from separated MRCC fractions and matched control cells from 860 patients with histologically proven, primary breast cancer diagnosed between 1983 and 1998 were analysed. Clinical follow-up including all medical data are completed for 596 patients. An observation time of at least 100 months after the time of first diagnosis is available for 353 patients. Patients with previous diagnosis of other malignant disorders as well as breast cancer patients with distant metastases at the time of first diagnosis were excluded. None of the patients received neo-adjuvant treatment before primary surgery. The median number of lymph nodes removed surgically was 18 (min 2; max 46). Median age of the patients (first diagnosis) was 51 years (range 25–85 years). Radiotherapy was given to 53% of the patients. 25% and 69% of the node-negative or -positive patients received adjuvant chemotherapy, respectively. 59% of all patients received hormonal therapy, either alone (34%) or in combination with chemotherapy (23%). During the observation time, 158/353 (44.7%) patients relapsed.

Further characteristics of patients and tumours are listed in Table 1. Blood samples derived from 24 apparently healthy female volunteers of our laboratory staff were used in control experiments. The patients participating in this study gave their informed consent for all analyses presented in this report.

**Table 1** Patients characteristics

| State | No relapse | Relapse within first 2 years after primary diagnosis | Relapse 2–5 years after primary diagnosis | Relapse more than 5 years after first diagnosis |
|---|---|---|---|---|
| Nodal state | | | | |
| N− (*n*, 146) | 92 (63%) | 26 (18%) | 19 (13%) | 9 (6%) |
| N+ (*n*, 161) | 82 (51%) | 37 (23%) | 30 (19%) | 12 (7%) |
| unknown (*n*, 46) | 21 (46%) | 8 (17%) | 11 (24%) | 6 (13%) |
| Tumour size | | | | |
| Tis (*n*, 5) | 3 (60%) | 1 (20%) | 1 (20%) | 0 (0%) |
| T1 (*n*, 125) | 85 (68%) | 17 (14%) | 16 (13%) | 7 (6%) |
| T2 (*n*, 156) | 78 (50%) | 37 (24%) | 28 (18%) | 13 (8%) |
| T3/4 (*n*, 30) | 13 (43%) | 9 (30%) | 7 (23%) | 1 (3%) |
| unknown (*n*, 37) | 16 (43%) | 7 (19%) | 8 (22%) | 6 (16%) |
| Grading | | | | |
| G1 (*n*, 17) | 10 (59%) | 3 (18%) | 3 (18%) | 1 (6%) |
| G2 (*n*, 136) | 79 (58%) | 27 (20%) | 16 (12%) | 14 (10%) |
| G3–4 (*n*, 112) | 59 (53%) | 23 (21%) | 27 (24%) | 3 (3%) |
| unknown (*n*, 88) | 47 (53%) | 18 (20%) | 14 (16%) | 9 (10%) |
| Menopausal | | | | |
| pre (*n*, 113) | 51 (45%) | 30 (27%) | 20 (18%) | 12 (11%) |
| post (*n*, 182) | 111 (61%) | 31 (17%) | 28 (15%) | 12 (7%) |
| unknown (*n*, 58) | 33 (57%) | 10 (17%) | 12 (21%) | 3 (5%) |

Clinical, pathological and biological status of 353 breast carcinoma patients stratified for nodal state (N), tumour size (T), grading (G) or menopausal state and time to relapse.

© 2000 Cancer Research Campaign 

### Isolation of minimal residual cancer cells (MRCC) and patient's matched control cells (Figure 1)

Peripheral blood was collected in heparinized Vacutainer systems (Becton Dickinson). Mononucleated cells (MNC) from 12.2 ml peripheral blood were purified over a density gradient using Nycoprep 1.077 (Nycomed, Norway). Collected MNC-fractions were washed twice with PBS (0.2% BSA; Life Technologies, Germany) and resuspended finally in 12.2 ml PBS (0.2% BSA). Cells derived from 5 ml of the MNC suspension were lysed in Trizol (Life Technologies, Germany) for archiving untouched material. 7 ml of the purified MNCs were used for subsequent isolation of MRCC. The cell suspension was separated by size using a column containing a polyester mesh (mesh-opening 20 μm; RELAB AG, Germany). After separation of the MNC suspension the mesh was rinsed 10 times using 5 ml PBS each. After washing, the cells retained on the mesh were lysed using Trizol (Life Technologies, Germany). Patient's matched CD45 positive lymphocytes were isolated from 0.2 ml of the MNC fraction using anti-CD45 mAb coated magnetic beads (Dynal, Norway) and lysed using Trizol. DNA and RNA of control cells and MRCC were extracted according to the manufacturer's instructions. Isolated DNA derived from MRCC fraction and from patients matched control lymphocytes were used to determine GI.

### Spiking and recovery of carcinoma cells

In spiking experiments, we used the SW480 colon carcinoma cell line SW480 because of its known *Ki-ras* codon 12 mutation (GGT (Gly) → GTT (Val)). SW480 tumour cells were grown for 24–48 hours either in silanized glass tubes leading to cellular aggregation or in normal tissue flasks using DMEM, 10% FCS as culture medium. Silanization of glassware was done using 1 ml/tube 0.5% Dimethyldichlorsilane in toluene (FLUKA, Germany). Glass tubes were incubated for 20 min and washed 3 times using 1.5 ml toluene each. Glass tubes were washed again using 1.5 ml methanol and finally using 5 ml 70% ethanol. Cells were harvested from tissue flasks using trypsin/EDTA solution (GibcoBRL, Germany) and washed twice (400 ***g***, 4˚C, 5 min) with PBS (0.2% FCS) leading in the majority to single cells. Cells from silanized glass tubes were transferred into a new glass tube and washed twice (400 ***g***, 4˚C, 1 min) with PBS (0.2% FCS). Cells were counted (an aliquot of clustered cells was previously trypsinized) using trypan blue exclusion. SW480 cells were spiked in 5 ml whole blood or in previously isolated peripheral blood mononuclear cells (MNC) derived from 5 ml heparinized blood as described. Reisolation of the spiked carcinoma cells and nucleic acid preparation of the resulting fractions was done as described above (Isolation of MRCC). The experiment was repeated three times. Analysis of *Ki-ras* codon 12 mutation and *DCC*-LOH was performed as described below.

### Analysis of 17-1A (EGP) expression

Analysis of 17-1A expression was done using RT-PCR. Briefly, RNA was isolated using Trizol and reversely transcribed using random-hexameres according to a standard protocol. The resulting cDNA was amplified using the following conditions:

EGP-1: 5′-6FAM-CGT CAA TGC CAG TGT ACT TC-3′
EGP-2: 5′-AAC GCG TTG TGA TCT CCT TC-3′

95˚C, 10 min/38 × (94˚C, 30s; 58˚C, 30s; 72˚C, 30s)/72˚C, 5 min.

The resulting FAM-labelled PCR product (390 bp) was analysed using capillary gel electrophoresis (Genescan genetic analyser 310 (AbiPrism, Perkin Elmer)).

### Analysis of genomic imbalances

*Ki-ras* codon 12 mutuation and *p53* hot-spot mutations were analysed using PCR with subsequent mutation-specific restriction of the PCR product (Chen et al, 1993; Hardingham et al, 1993). Quantification of the restricted PCR products was done using capillary gel electrophoresis (Genescan genetic analyser 310 (AbiPrism, Perkin Elmer)).

Patterns of allelic losses and amplified oncogenes were measured for *p53* (*TP53*), *RB-1* (exon 20), *DCC* and *APC* (McGee et al, 1989; van Leeuwen et al, 1991; Huang et al, 1992; Cawkwell et al, 1994; Kirchweger et al, 1994; Cho et al, 1996; Crundwell et al, 1996). Furthermore, GI were detected using the following microsatellite specific primers; D17S926 (AFM207xa11), D17S695 (nm00089), D17S849 (AFM234wg3), D17S960 (AFMa-120xd5), D16S265 (D16S265.PCR1), D16S496 (AFM214ZG5) and D11S528 (42026/27) (Hauge et al, 1991; Carter et al, 1994; Gyapay et al, 1994) and *p16* on regions D9S126 (D9S126. PCR1) and D9S171 (AFM126xc3) (Liggett and Sidransky, 1998; Villuendas et al, 1998). Amplification of oncogenes was measured using a multiplex PCR using primer sequences specific for *c-erbB-2* or *c-myc* coamplified with *β-globin*, distinguishable both by fluorescence label and size. Sense primers were labelled with fluorogenic molecules 6-FAM or HEX (TIBMOLBIOL, Germany). Briefly, DNA-PCR products resulting from CD45 positive lymphocytes (wild-type control) were compared with the PCR profile generated from the isolated MRCC-fraction. Resulting products were analysed by capillary gel electrophoresis (Genescan genetic analyser 310; AbiPrism, Perkin Elmer). All PCR parameters were optimized for reliable amplification and fluorogenic detection (further information can be requested from the authors). To determine loss of heterozygosity allele ratios were calculated as described (Cawkwell et al, 1993) with minor alterations. Briefly, the size of both alleles for heterozygous (informative) patients was assigned according to both peaks of greatest integral in the patients CD45 positive control sample. The peak integrals of the two alleles in the paired normal and tumour sample were used to determine genomic imbalance (GI) using a relative allelic difference of 50% as a cut-off value. Furthermore, the presence of LOH was accepted as positive only, if the integral of the resulting microsatellite peak was ≥2000 and the peak/noise ratio was ≥50.

In case of amplified oncogenes *c-myc* and *c-erbB-2* GI was calculated by the value of the peak area derived from the analysed oncogene compared with the integral of the β-globin peak: OT:GT/ON:GN, where OT and ON are the integrals of the oncogene peak areas and GT and GN the peak area values of β-globin for the tumour (T) and CD45 control sample (N). Genomic imbalance (amplification) was given if the resulting ratio was greater than or equal 2.00. Furthermore, the presence of an amplification was accepted as positive only, if the integral of the resulting PCR-product peak was ≥2000 and the peak/noise ratio was ≥50.

 *© 2000 Cancer Research Campaign*

### Statistical analysis

To compare the frequencies of categorical variables between two groups the Chi-Square test was used, with $P < 0.05$ considered as statistically significant. The relationship between the dependent variable 'genomic imbalance' of DNA and the independent variables nodal status, menopausal status and grading was assessed by use of a logistic regression. Relapse-free survival interval (RFS) was defined as the period between date of first diagnosis of breast cancer and documented relapse. Patients without an event were censored. Survival curves were drawn with the use of Kaplan–Meier product limit method. The log-rank test was used to compare two or more survival distributions. Multivariate analyses were performed using the Cox proportional hazards model, *P* values were derived from Wald's Chi-Square test. To the basic multivariate model, which included nodal status, menopausal status and grading, the variable 'genomic imbalances' of DNA was added. The important prognostic factors were identified by use of the stepwise selection method. All computations were performed using the SAS software package, release 6.12.

### RESULTS

### Spiking and recovery of carcinoma cells

The proof of principle of cancer cell separation by size was performed using the colon carcinoma cell line SW480 because of its known *Ki-ras* codon 12 mutation. Recovery and purity of the re-isolated tumour cells after spiking of single and aggregated SW480 carcinoma cells in previously isolated mononuclear cells was determined (Table 2). The majority of aggregated cells generated after growing them for 24 hours in silanized glass tubes showed clusters consisting of 5 to 15 cells 30–70 μm in size (Figure 2B). In contrast, the majority of trypsinized SW480 cells consisted of single cells with 16.5 μm in diameter (Figure 2A). Purity and recovery of spiked and reisolated SW480 single and clustered cells was determined by the analysis of *Ki-ras* codon 12 mutation using a restriction-fragment-length-polymorphism analysis (RFLP). Using this kind of analysis, a fragment of 114 bp is indicative for wild-type *Ki-ras* codon 12, whereas a product of 143 bp resembles a mutated *Ki-ras* codon 12. Analysing the two peak integrals resulting from capillary electrophoresis, one is able to determine the relative distribution of mutated vs wild-type fragments. Whereas the mean recovery of clustered cells varied between 54% and 68%, the mean purity was generally higher than 50%. In case of single cells positive results could not be obtained.

Amplification of 17-1A specific RNA from isolated aggregated SW480 cancer cells showed strong positive signals, but only a weak signal in the isolated fraction when 1500 single cells were spiked. In parallel, microsatellite analysis of the DCC tumour suppressor gene revealed reliable detection of 'LOH' after spiking and isolation of 30 aggregated SW480 cells (data not shown).

### Genomic imbalances in minimal residual cancer cells isolated from blood of breast cancer patients

In MRCC fractions derived from 353 breast cancer patients (Table 1) and 24 healthy volunteers we analysed the following genes and chromosomal regions: amplification of *c-erbB-2* and *c-myc*, *p53*-mutations (5 hot-spot mutations) and LOH of *p53*, *DCC*, *RB-1*, *APC*, D9S126, D9S171, D16S496 and D17S695. Using this panel of analyses, we neither found GI of either tumour suppressor genes nor proto-oncogenes in MRCC fractions derived from 24 healthy volunteers (data not shown). In breast cancer patients ($n = 353$) we found MRCC with several GI in different genes and chromosomal regions. When these patients were stratified into 4 groups with respect to GI of the *DCC* and *c-erbB-2* gene (Figure 3; I, III, IV and V), amplification of the proto-oncogene c-erbB-2 and LOH of the DCC gene showed a clear and significant correlation with a worse RFS (I vs III: $P = 0.006$; I vs V: $P = 0.0004$). These differences were also significant if compared with the actuarial RFS of

**Table 2** Reisolation of spiked carcinoma cells

| Number of spiked cells | Analysis of Ki-ras Codon 12 mutation[a] Integral 114 bp (wt) | Integral 143bp (mt) | Recovery[b] (%) | Purity[c] (%) | 17-1A[d] Integral 390 bp |
|---|---|---|---|---|---|
| agg 1500 | 8410 ± 2741 | 23322 ± 7054 | 64 ± 12 | 73 ± 5 | 19846 ± 2106 |
| agg 300 | 2730 ± 1996 | 6981 ± 2580 | 57 ± 9 | 75 ± 16 | 7798 ± 1142 |
| agg 150 | 2969 ± 2586 | 4760 ± 2010 | 68 ± 13 | 66 ± 13 | 5276 ± 1327 |
| agg 30 | 1480 ± 1083 | 1562 ± 1504 | 54 ± 17 | 50 ± 14 | 3609 ± 953 |
| 0 | 1583 ± 456 | nd | – | – | nd |
| sin 1500 | 7309 ± 1634 | nd | nd | nd | 547 ± 340 |
| sin 300 | 4031 ± 882 | nd | nd | nd | nd |
| sin 150 | 2192 ± 896 | nd | nd | nd | nd |
| sin 30 | 5003 ± 3700 | nd | nd | nd | nd |
| 0 | 3197 ± 2927 | nd | – | – | nd |
| Input control | | | | | |
| 1500 SW480 | nd | 39769 ± 15635 | – | 100 | |
| 300 SW480 | nd | 13240 ± 5553 | – | 100 | |
| 150 SW480 | nd | 6534 ± 2224 | – | 100 | |
| 30 SW480 | nd | 2324 ± 1641 | – | 100 | |

Aggregated (agg) and single (sin) SW480 colon-carcinoma cells were spiked in MNCs derived from 5 ml heparinized blood from healthy volunteers and reisolated by size as described. The values correspond to the average ± standard deviation of the peak integrals of the PCR-products derived from 3 independent experiments. [a]Ki-ras codon 12 mutation was analysed by PCR with subsequent mutation-specific restriction of the PCR product as described in Materials and methods leading to a 114 bp fragment (wild-type) or to a 143 bp fragment (mutated). [b]Recovery was calculated using the integral of the SW480 input control as 100%.
[c]Purity was calculated by addition of both peak integrals (114 bp + 143 bp) = 100%. [d]The expression of the 17-1A (EGP) antigen was semiquantitatively measured using a RT-PCR system as described. nd: not detected; sin: single cells; agg: aggregated cells.

© 2000 Cancer Research Campaign 

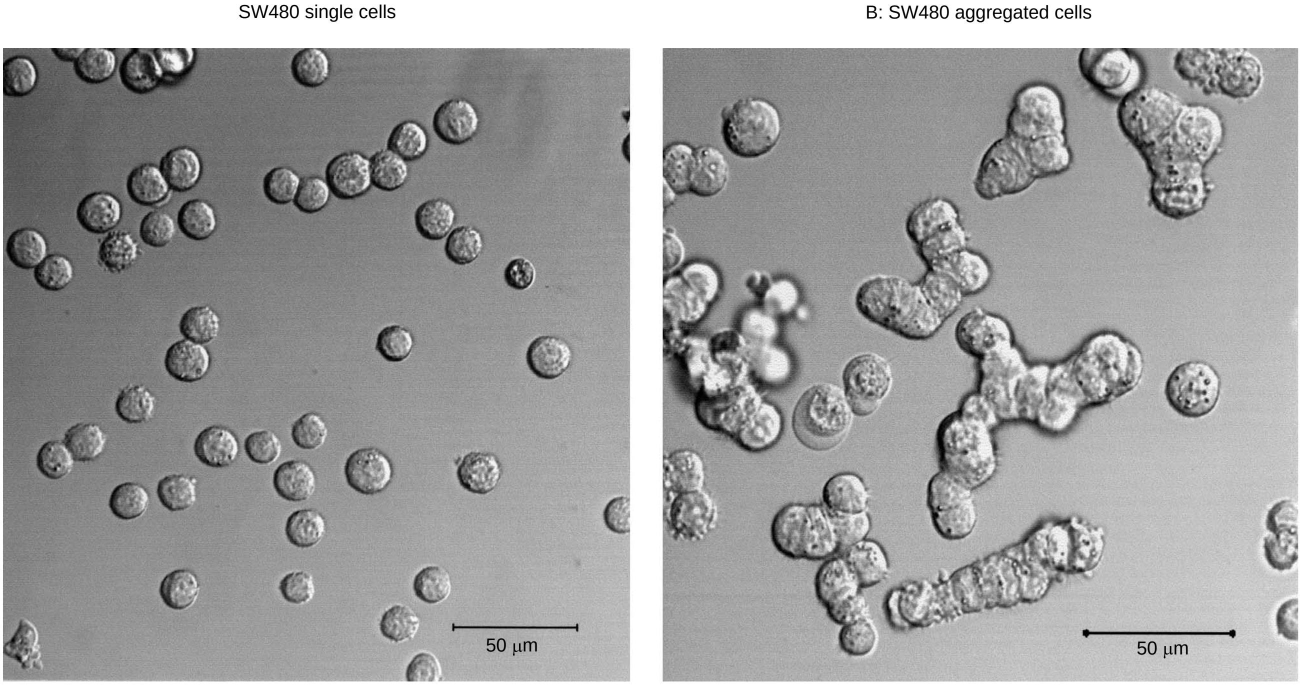

**Figure 2** Comparison of aggregated vs not aggregated SW480 carcinoma cells. Cells were grown for 24–48 hours in tissue flasks (RPMI/FCS) and harvested using trypsin/EDTA leading in a majority to single cells (**A**). For aggregation the cells were grown in silanized glass tubes and harvested after 24–48 hours of incubation (**B**). The majority of cells formed aggregates ranging from 5 to 15 cells. The bar represents 50 μm

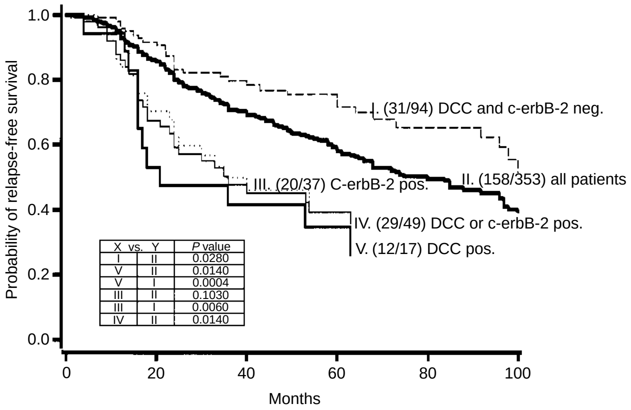

**Figure 3** Actuarial relapse-free survival curves for breast cancer patients stratified by genomic imbalances of *c-erbB-2* and *DCC* in isolated MRCC. MRCC fractions from all patients ($n$ = 353; II: RFS for all, unstratified patients) were analysed. Patients tested positive for *c-erbB-2* amplification (III), *DCC*-LOH (V) and positive for GI of *c-erbB2* or *DCC* (IV) were given. Patients tested negative (I) were included only, if the analysis was informative (heterozygous; 60%) for *DCC* and evaluable (peak-integrals of the measured PCR-products) for *c-erbB-2* and *DCC* analyses as described in the Materials and methods section. Numbers between brackets: failures/total number of patients in each group. Statistical analyses are shown in the box unstratified (all) patients (Figure 3, IV vs II; $P$ = 0.014). Figure 4 shows the actuarial RFS curves of patients as a function of GI (14 parameters) (A), nodal status (B) and grading (C). Clearly, patients with ≥2 detectable GI in MRCC (Figure 4A) experienced a very poor prognosis. We found a clear and significant correlation comparing the number of GI detected in MRCC and the actuarial RFS (all patients vs ≥2 GI: $P$ = 0.026; 0 GI vs ≥2 GI: $P$ = 0.0008). In turn, patients with no detectable GI in the MRCC fraction encountered a favourable prognosis (0 GI vs all: $P$ = 0.021). Analysing RFS as a function of nodal status or grading (Figure 4 B,C), significant differences were found only between nodal negative and positive patients ($P$ = 0.026). In Figure 5 we plotted the actuarial RFS of nodal negative and nodal positive patients as a function of GI (14 parameters) in MRCC. When dividing nodal negative patients into two groups by the number of GI identified in MRCC (I, 0–1 GI and III, ≥2 GI), increasing numbers of GI were significantly correlated with poor prognosis of an ongoing disease (0–1 GI vs ≥2 GI: $P$ = 0.001). In nodal positive patients (Figure 5B) we also found a clear correlation of the number of GI in MRCC and worse RFS (0–1 GI vs ≥2 GI: $P$ = 0.001; all patients vs ≥2 GI: $P$ = 0.016). On the other hand, we found an improved RFS associated with lower numbers of GI (especially negative for MRCC if no GI was detected) in MRCC (0–1 GI vs all patients: $P$ = 0.034).

Analysing the actuarial RFS in all patients without adjuvant chemotherapy (Figure 6A; $n$ = 178), we also found a clear and significant correlation comparing the number of GI detected in MRCC and the actuarial RFS (0–1 GI vs ≥2 GI; $P$ = 0.006). In nodal negative patients analysed before adjuvant chemotherapy ($n$ = 92 from 596) for GI in MRCC we found in 23% more than or equal 2 GI (Figure 6B). On the other hand, 77% of these patients tested were either negative (33%) or positive for only one GI (44%) in separated MRCC.

Analysing MRCC isolated from $N_0M_0$ patients, we found 0, 1 and ≥2 GI in 21.8%, 43.6% and 34.5% of the patients samples, respectively. Whereas 16% of the patients without detectable GI in isolated MRCC relapsed, 55% of the patients with ≥2 GI showed an ongoing disease ($P$ = 0.003) (Figure 6C, $n$ = 110). To clearly verify that increasing numbers of GI detected in disseminated cancer cells of patients correlates with worse prognosis of clinical outcome, we re-analysed 6 additionally GI in separated MRCC derived from blood of 199, randomly selected patients (Figure 7; 20 parameters; resulting from 353 patients with complete medical

 *© 2000 Cancer Research Campaign*

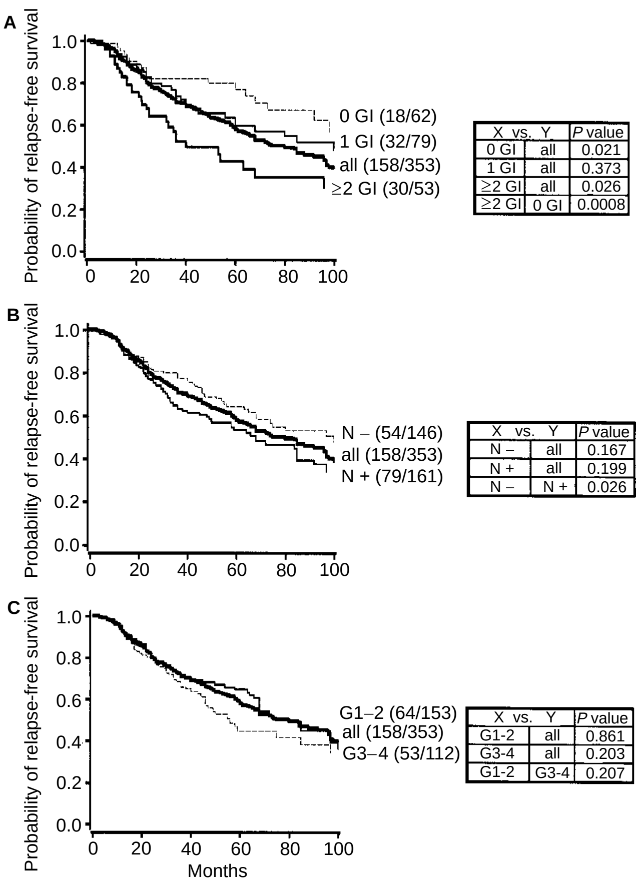

**Figure 4** Actuarial relapse-free survival curves for breast cancer patients stratified by genomic imbalances detected in isolated MRCC of the blood (**A**), nodal status (**B**) and grading (**C**). The following genes and chromosomal regions were analysed (14 parameters: *c-erbB-2* and *c-myc* amplification, *p53*-mutation (codon 175, 245, 248, 249, 273), *DCC*-, *RB-1*-, *APC*-, *p53*-, D9S126, D9S171, and D17S695). In plot A, MRCC from all patients ($n$ = 353, thick line: RFS for all, unstratified patients) were analysed. Patients tested positive for genomic imbalances were included as indicated (1 GI and ≥2 GI). Patients tested negative for GI in MRCC were included only if the analysis was informative (heterozygous) for DCC and evaluable (peak-integrals of the measured PCR-products) in c-erbB-2 and DCC analyses as described in the Materials and methods section. Numbers between brackets: failures/total number of patients in each group. Statistical analyses are shown in the box

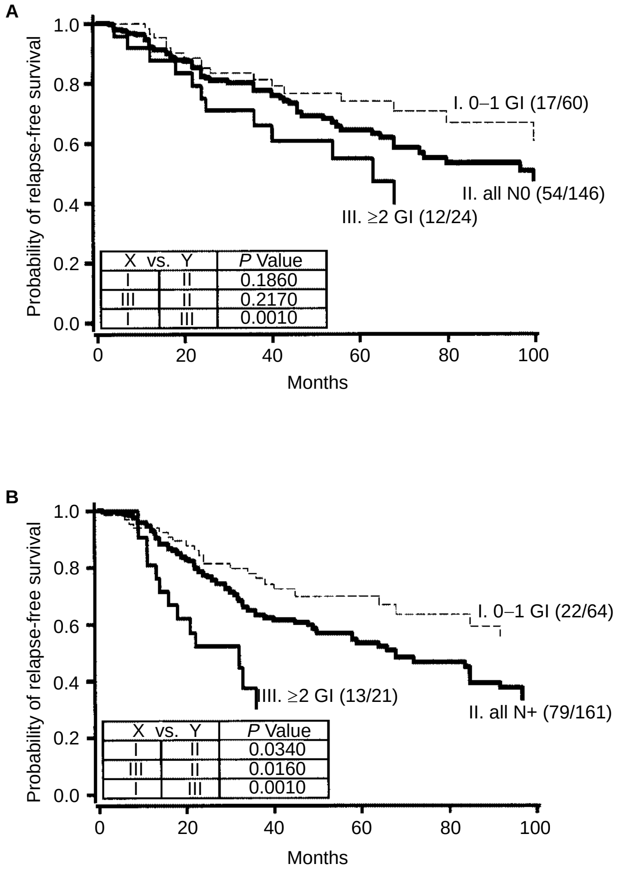

**Figure 5** Actuarial relapse-free survival curves for breast cancer patients stratified by genomic imbalances (14 parameters; see Figure 4) detected in isolated MRCC of the blood derived from nodal-negative (**A**; $n$ = 146) and nodal-positive (**B**; $n$ = 161) patients. Patients tested positive for genomic imbalances in isolated MRCC were included as indicated (1 GI and ≥2 GI). Patients tested negative for GI in MRCC were included only if the analysis was informative (heterozygous) for *DCC* and evaluable (peak-integrals of the measured PCR-products) in *c-erbB-2* and *DCC* analyses as described in the Materials and methods section. Line II: RFS for all, unstratified patients within this group. Numbers between brackets: failures/total number of patients in each group. Statistical analyses are shown in the box files and full observation time). In control samples, no GI could be detected using these marker panel (data not shown). Again, patients without detectable GI in the MRCC fraction were used in calculations only, if the DCC marker was informative and analysis of DCC and c-erbB-2 was reliable, leading to 183 patients analysed for actuarial RFS. In accordance with our previous results, we found a clear correlation between increasing numbers of GI measured in MRCC and worse RFS. There was a tendency, not a statistically significant difference, comparing actuarial RFS of patients without detectable GI in MRCC and patients with 1 GI in isolated cancer cells. Comparing patients with no detectable GI in MRCC and patients with 2–3 GI or ≥4 GI in disseminated cancer cells, we found again highly significant statistical differences in RFS with $P$ = 0.004 and $P$ = 0.002, respectively.

The quantity of GI in MRCC of 183 patients evaluable for analysis of relapse-free survival ranged from 1 to 7. In logistic regression (Table 3A) including GI, nodal status, grading and menopausal status, we found GI as an independent factor ($P$ value ranged from 0.40–0.73). Cox multivariate regression analysis was performed to compare the prognostic significance of GI in MRCC with that of the classical prognostic parameters (Table 3B), these latter variables comprising a basic multivariate model. Corrected for the basic multivariate model, GI in MRCC were highly related with a poor RFS ($P$ = 0.002).

### DISCUSSION

In addition to the classical prognostic factors for patients with breast cancer, it would be beneficial to have tools available, which could independently predict the recurrence of disease of individual patients, define possible drug targets and to follow up the efficacy of response to systemic therapy. Consequently many research groups have done considerable work to detect or isolate disseminated cancer cells. In most cases, the isolation procedures based on immunological methods. Paramagnetic beads conjugated with monoclonal antibodies were used to capture disseminated (epithelial) cells (Brandt et al, 1998; Soria et al, 1999). Major limitations of immunomagnetic isolation protocols are altered or masked antigenic epitopes and down-regulation of antigen expression.

© 2000 Cancer Research Campaign 

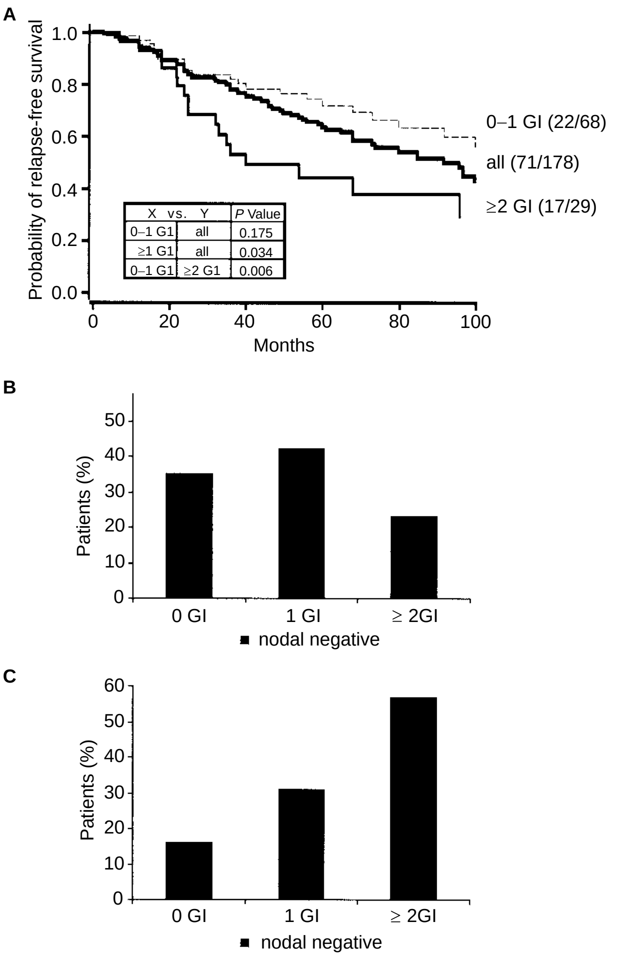

**Figure 6** (**A**) Actuarial relapse-free survival curves for breast cancer patients stratified by genomic imbalances (14 parameters; see Figure 4) detected in isolated MRCC of the blood derived from all patients receiving no adjuvant chemotherapy (*n* = 178; thick line: RFS of all, unstratified patients). Patients tested positive for genomic imbalances in isolated MRCC were included as indicated (0–1 GI and ≥2 GI). Patients tested negative for GI in MRCC were included only if the analysis was informative (heterozygous) for *DCC* and evaluable (peak-integrals of the measured PCR-products) in *c-erbB-2* and *DCC* analyses as described in the Materials and methods section. Numbers between brackets: failures/total number of patients in each group. Statistical analyses are shown in the box. (**B**) Comparison of the number of GI (14 parameters; see Figure 4) detected in isolated MRCC from nodal-negative patients before adjuvant chemotherapy (*n* = 92/596).
(**C**) Comparison of the number of GI (14 parameters, see Figure 4) detected in isolated MRCC from $N_0$ $M_0$ patients (*n* = 110; 0 GI: 24 (21.8%); 1 GI: 48 (43.6%); ≥2 GI: 38 (34.5%)) and the clinically diagnosed relapse during the observation period (0 GI: 4/24 (17%), 1 GI: 15/48 (31%), ≥2 GI: 21/38 (55%); 0 GI vs 1 GI: *P* : not significant; 0 GI vs ≤ 2 GI: *P* = 0.003)

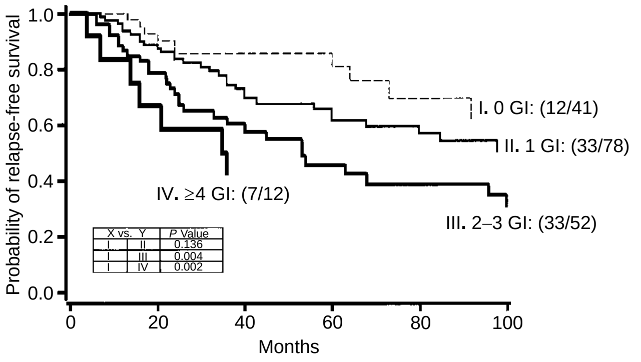

**Figure 7** Actuarial relapse-free survival curves for breast cancer patients stratified by genomic imbalances (20 parameters; see Figure 4 and D11S528, D16S265, D16S496, D17S960, D17S849, D17S926) detected in isolated MRCC of the blood (*n* = 183/199). Patients tested positive for genomic imbalances in isolated MRCC were included as indicated (1 GI, 2–3 GI and ≥4 GI). All patients without detectable GI in MRCC fraction were used only, if analyses were informative (heterozygous) for *DCC* and evaluable (peak-integrals of the measured PCR-products) in *c-erbB-2* and *DCC* analyses as described in the Materials and methods section. Numbers between brackets: failures/total number of patients in each group. Statistical analyses are shown in the box

Furthermore, the basal transcription of epithelial specific antigens in normal blood cells (de Graaf et al, 1997) may affect the purity of isolated tumour cells.

We developed a novel technique designed for a continuous monitoring of cancer management in individual patients. In contrast to antigen-dependent purification, cells larger than 20 μm and cancer cell aggregates, eventually micrometastases, were isolated through a filtration process. Because the outcome of this procedure is susceptible for aggregated normal cells, we optimized our isolation protocol preventing passive aggregation. The first and crucial step in density and size dependent MRCC purification is blood collection and isolation of MNCs using a density gradient centrifugation. Normally, blood samples are collected in plastic syringes containing anticoagulants, systems which are in general susceptible for micro-agglutination. Furthermore, isolation of MNCs is often performed using density media based on Ficoll. These synthetic polysaccharides lead to an aggregation of red blood cells and possibly to a clotting of rare cells with or within the aggregated blood cells. To overcome these problems of passive aggregation we used heparinized glass vacutainer systems for blood collection. Furthermore, the density medium NycoPrep does not contain polysaccharides thus preventing the generation of aggregates during MNC isolation. Using these modifications, MNC fractions without visible aggregates could be obtained.

To prove the principle of our isolation system, we used MNCs of healthy donors spiked with aggregated or single SW480 colon carcinoma cells (Figure 2 and Table 2). SW480 colon carcinoma cells were used because of the known *Ki-ras* codon 12 mutation which is readily detectable by PCR with subsequent mutation-specific restriction of the PCR product. In first experiments we spiked single and clustered tumour cells in whole blood of healthy volunteers. Despite good recovery of the spiked SW480 cells using size-dependent separation, the purity was generally lower than 50%. Further analyses of the isolated cells using cytospins and FACS showed tumour cells aggregated with $CD45^+$ blood lymphocytes (data not shown). This can be explained by the allogeneic differences of the spiked SW480 cells and donor lymphocytes, circumstances not given in real patient samples. Therefore, to reduce the time leading to lymphocyte–tumour cell interactions we used previously isolated MNC from healthy volunteers in reisolation experiments. Using a simple but effective filtration process it could be observed that MNCs (normally <18 μm) and single SW480 carcinoma cells passed the mesh, whereas cells/aggregates larger than 20 μm were retained. The success of this physical process was shown by the analysis of *Ki-ras* mutation. In contrast to single cells, aggregated cells were isolated with a purity higher than 50% which enables analysis of GI at the DNA level. On the other hand, we frequently found relatively high peak-integrals corresponding to the wild-type fragment in the spiking assays using single SW-480 cells. Nevertheless, the wt-integrals were within the standard deviation obtained for

 © 2000 Cancer Research Campaign

**Table 3** Statistical assessment of the independent prognostic value of GI

**A** Logistic regression

| | | **Responses/levels** | ***P* value for $H_0$: β = 0** |
|---|---|---|---|
| Dependent variable | GI | 0 or 1 / ≥2 | |
| Independent variables | nodal status | negative/positive | 0.42 |
| | grading | G1 or G2/G3 or G4 | 0.73 |
| | menopausal status | pre/post | 0.40 |

**B** Cox multivariate analyses of relapse-free survival

| **Factor** | ***P* value** | **RHR** | **(95% CI)** |
|---|---|---|---|
| Nodal status | 0.13 | 1.60 | 0.87–2.97 |
| Grading | 0.25 | 1.43 | 0.77–2.65 |
| Menopausal status | 0.07 | 0.56 | 0.31–1.04 |
| GI in purified MRCC | 0.002 | 1.52 | 1.16–1.98 |

Relative hazard rate (RHR) and 95% confidence interval (CI) of multivariate analysis (final model, patients corrected for the basic model, including nodal status, grading and menopausal status; $n$ = 183).

aggregated cells. The background of measured wt-integrals with the observed differences may be explained by a passive adhesion of ‘normal’ blood lymphocytes at the mesh. In contrast, wt-integrals in spiking assays using single SW-480 cells are somewhat higher if compared with assays using aggregated SW-480 tumour cells. We cannot exclude that the different growing conditions (silanized glass tubes vs. tissue flasks) will have any effect on the expression and function of e.g. adhesion molecules. Furthermore, single cells used in our assays had been trypsinized, whereas aggregated cells were not treated. These different treatments may lead to the slightly higher values of wt-integrals in single cell assays. On the other hand, this assay acts as a ‘proof of principle’ and showed clearly the possibility of size-dependent isolation of aggregated tumour cells. The highly selective purification of spiked aggregated tumour cells was further shown using RT-PCR specific for the 17-1A (EGP) antigen. In whole blood and isolated MNC, EGP-specific RT-PCR always leads to positive results (de Graaf et al, 1997 and own observations). Contrastingly, positive results were not found in MRCC-fractions derived from 24 healthy volunteers, showing clearly the possible applicability of RT-PCR analyses from purified MRCC.

The methodical evaluation indicated an extremely high diagnostic specificity of MRCC detection using DNA analyses. On the other hand, molecular detection and characterization of purified MRCC from patients has to reflect the polyclonality of malignant disorders (Aubele et al, 1999). Furthermore, GI in metastases and MRCC are quite different from alterations found in the primary tumour (Hampl et al, 1999; Offner et al, 1999). Therefore, single parameter analyses as performed by many scientific groups, does not describe the complexity of this disease. To overcome these problems, specific analytical proof for the presence and characterization of MRCC was performed at the DNA level measuring up to 20 GI. This panel was necessary to increase the diagnostic sensitivity. Using this full panel of analyses (Figure 7), we found 71% of all patients analysed positive for MRCC defined by at least one GI. The detection rate of MRCC was not associated with nodal status or grading of the primary tumour. Contrastingly, stratification of patients by genomic characterization of MRCC had an enormous prognostic impact. The best prognostic value within our analytical panel was provided by GI of the *DCC* and *c-erbB-2* genes (Figure 3). Determination of these two GI led to the clear identification of 15.2% of the patients with worst prognosis and 26.6% of all patients encountered a favourable prognosis of RFS. Whereas the nodal status of patients (307/353 with known nodal status) provides prognostic information on RFS (Figure 4B; $P$ = 0.026), evaluation of grading (Figure 4C; 265/353) did not reach statistic significance. Contrastingly, analyses of GI (14 parameter) in isolated MRCC (Figure 4A) showed a highly significant correlation with respect to RFS (≥2 GI vs 0 GI; $P$ = 0.0008). Because informative analyses of DCC and c-erbB-2 were extremely relevant, we stated patients as negative for MRCC (0 GI) only, if both analyses were informative and reliable. Using a panel of 14 analyses, we increased the diagnostic sensitivity of MRCC detection defined by one or more GI found in disseminated tumour cells.

There was only a tendentious, but not significant correlation in RFS from patients with only one GI in MRCC compared with MRCC negative (no detectable GI) patients. In contrast with recently published data (Engel et al, 1999), we found a clear association of increasing numbers of GI found in MRCC with worse prognosis, a model also discussed earlier for primary tumours (Harada et al, 1994). This correlation was independent from nodal status (Figure 5) and grading (data not shown) of the patients primary tumour. Determining possible effects derived from chemotherapeutic treatment on MRCC in blood, we stratified patients without adjuvant chemotherapy. Again we found remarkable and significant correlations comparing the number of GI in MRCC with RFS (Figure 6A). Furthermore, in nodal-negative patients analysed before adjuvant chemotherapy (Figure 6B; 92/596), we found in 65% MRCC; 23% of these patients were positive for MRCC with ≥2 GI. These patients may belong to a clearly defined high-risk group experiencing benefit from chemotherapeutic intervention. This assumption could further be confirmed if considering the fate of the $N_0M_0$ patients (Figure 6C). An ongoing disease was significantly associated with increasing GI measured in MRCC. Therefore, purification and genotyping of MRCC should allow individualized therapeutic decisions in nodal-negative patients, a subgroup of patients where therapeutic decisions and prognostication of ongoing disease is extremely difficult.

To further increase the diagnostic sensitivity and therefore, leading to a better characterization of MRCC derived from the

© 2000 Cancer Research Campaign 

blood of breast cancer patients, we re-analysed in 199/353 randomly selected patients 6 additional chromosomal regions for the presence of GI (Figure 7). Again, all healthy volunteers tested were negative for these GI in isolated cell fractions. Therefore, we assume a nearly 100% specificity of our analyses (data not shown). In these 199 patients we analysed 20 chromosomal regions. Using this panel of parameters, the detection rate of MRCC was 71% of the patients with ≥1 GI in MRCC. Again, increasing numbers of GI in MRCC were highly correlated with worse RFS (0 GI vs ≥4 GI: $P = 0.002$).

The interesting finding of the present study concerns the individualized prognostic impact of increasing GI in isolated MRCC from blood of breast cancer patients. Furthermore, logistic regression and multivariate analyses (Table 3) favoured GI, independent from nodal status, grading and menopausal status, as the best prognostic factor. Contrasting to analyses of the primary tumour, MRCC detection and characterization enables continuous monitoring of patients.

Using this system, we identified in nearly 71% of the patients (Figure 7, 20 parameters) MRCC with one or more GI. On the other hand, highly statistical significance with respect to RFS was given only if 2 or more GI were present in MRCC. The same holds true if the patients were analysed using the reduced set of 14 parameters but with a lower overall detection rate of MRCC. Due to the heterogeneity of tumours, an increase in the number of analytical parameters leads consequently to an increased sensitivity in MRCC detection. These findings may be explained by the existence of different cancer cell clones in the blood of breast cancer patients. We hypothesize that dissemination of cancer cells from the primary tumour occurs in nearly all patients, leading to the definition of cancer spread as a systemic disease. The total number of GI accumulated within the tumour cells seemed to be crucial for the estimation of individual risk for early recurrence of disease. Despite the high percentage of MRCC positive patients these data are concordant with recently published data. Soria et al (1999) detected telomerase positive cancer cells using RT-PCR after immunomagnetic purification of 17-1A antigen expressing MRCC in 84% of advanced breast cancer patients (Stage IV). Brandt and co-workers (1998) found cytokeratin positive MRCC using immunohistochemistry in 63% of the blood samples from breast cancer patients at different stages. Furthermore, they found predominantly aggregated cytokeratine-positive tumour cells. Single and clustered cells were found in 45% and 83% of the positive patients, respectively and c-erbB-2 amplification could be detected more often in aggregated than in single tumour cells. For these reasons, the quoted authors favoured the hypothesis of two different, morphologically and genomically (c-erbB-2 amplification) distinguishable MRCC subpopulations in the blood. They hypothesize that aggregated tumour cells are a prerequisite for metastases. Using our system, we are not only able to identify MRCC in the blood of breast cancer patients; further characterization of GI using multiple markers of chromosomal alterations will give insight into tumorigeneic processes leading to metastases and probably into tumour biological behaviour. We do not believe that the high incidence of MRCC detection was a result of false positive findings. The isolation and detection system is extremely sensitive and reliable for the following reasons: (1) the analysis of GI is independent from background transcription often called illegitimate transcription of tumour-specific or -associated antigens, problems often described in RT-PCR analyses; (2) the cut-off value of 50% relative allelic difference in determining LOH or amplification was set extremely high; (3) there is no predominant loss of the 'longer' allele amplificate resulting after microsatellite PCR; (4) we never found GI in MRCC-fractions derived from healthy volunteer control samples, showing the specificity of the diagnostic system; (5) applying multi-parameter analysis of GI in separated MRCC will increase the sensitivity of the detection system.

Tumour cells in metastases do not reflect the genotype of the matched primary tumour (Hampl et al, 1999). Therefore, we compared GI in MRCC with alterations published for primary tumours. Kirchweger et al (1994) described LOH of *TP53* in 46% of breast cancer specimens analysed. Using our method, we identified MRCC with *p53* LOH in only 16% of all informative cases ($n = 245/353$) favouring the hypothesis of major genomic differences between the primary tumour and MRCC. This fact is further substantiated by data published from Offner et al (1999) for p53 overexpression. Whereas Carter et al (1994) found LOH of D11S528 in 39% of primary tumour samples investigated, MRCC showed LOH in nearly 20% of all informative cases ($n = 205/353$). These observations show clearly that disseminated cancer cells do not reflect the genotype of the primary tumour. These differences may be explained by the heterogeneity of primary tumours leading to extravasation of distinct subclones and/or by additional mutational events occurring in MRCC during circulation and retention in different organs, leading eventually to sessile micrometastases and recirculation (Aubele et al, 1999; Hampl et al, 1999). Accumulation of GI during circulation and retention of MRCC may be a result of clonal selection altering the tumour biologic behaviour. On the other hand, sessile tumour cells within the primary tumour accumulate other GI leading to distinct genomic differences comparing primary carcinomas and MRCC.

Summarizing our data we present a new, density- and size-dependent isolation method for the identification and characterization of disseminated tumour cells from peripheral blood of breast cancer patients. Isolation of large cells and aggregates leads to a cellular fraction of MRCC with detectable GI. The detection of disseminated tumour cells, especially the quantity and the composition of genomic imbalances correlated with the clinical outcome of the patients. Analyses of relapse-free survival and the quantity of GI showed the independent prognostic impact generated by genotyping of MRCC. The major genomic differences between primary tumours and MRCC favours the analysis of MRCC in the blood as the real target for systemic therapy. This system enables not only the detection and characterization of minimal residual disease of individual patients, it seems also very useful to estimate prognosis, progression and prediction of cancer patients leading to a new approach in cancer management.

### REFERENCES

Aigner S, Sthoeger ZM, Fogel M, Weber E, Zarn J, Ruppert M, Zeller Y, Vestweber D, Stahel R, Sammar M and Altevogt P (1997) CD24, a mucin-type glycoprotein, is a ligand for P-selectin on human tumor cells. *Blood* **89**: 3385–3395

Al-Mehdi AB, Tozawa K, Fisher AB, Shientag L, Lee A and Muschel RJ (2000) Intravascular origin of metastasis from the proliferation of endothelium-attached tumor cells: a new model for metastasis. *Nat Med* **6**: 100–102

Aubele M, Mattis A, Zitzelsberger H, Walch A, Kremer M, Hutzler P, Hofler H and Werner M (1999) Intratumoral heterogeneity in breast carcinoma revealed by laser-microdissection and comparative genomic hybridization. *Cancer Genet Cytogenet* **110**: 94–102

 *© 2000 Cancer Research Campaign*

Brandt B, Roetger A, Heidl S, Jackisch C, Lelle RJ, Assmann G and Zanker KS (1998) Isolation of blood-borne epithelium-derived c-erbB-2 oncoprotein-positive clustered cells from the peripheral blood of breast cancer patients. *Int J Cancer* **76**: 824–828

Carter SL, Negrini M, Baffa R, Gillum DR, Rosenberg AL, Schwartz GF and Croce CM (1994) Loss of heterozygosity at 11q22-q23 in breast cancer. *Cancer Res* **54**: 6270–6274

Cawkwell L, Bell SM, Lewis FA, Dixon MF, Taylor GR and Quirke P (1993) Rapid detection of allele loss in colorectal tumours using microsatellites and fluorescent DNA technology. *Br J Cancer* **67**: 1262–1267

Cawkwell L, Lewis FA and Quirke P (1994) Frequency of allele loss of DCC, p53, RBI, WT1, NF1, NM23 and APC/MCC in colorectal cancer assayed by fluorescent multiplex polymerase chain reaction. *Br J Cancer* **70**: 813–818

Chen PH, Lin SY, Wang CK, Chen YJ, Chen TC and Chang JG (1993) “Hot spots” mutation analysis of p53 gene in gastrointestinal cancers by amplification of naturally occurring and artificially created restriction sites. *Clin Chem* **39**: 2186–2191

Cho JH, Noguchi M, Ochiai A and Hirohashi S (1996) Loss of heterozygosity of multiple tumor suppressor genes in human gastric cancers by polymerase chain reaction. *Lab Invest* **74**: 835–841

Crundwell MC, Chughtai S, Knowles M, Takle L, Luscombe M, Neoptolemos JP, Morton DG and Phillips SM (1996) Allelic loss on chromosomes 8p, 22q and 18q (DCC) in human prostate cancer. *Int J Cancer* **69**: 295–300

de Graaf H, Maelandsmo GM, Ruud P, Forus A, Oyjord T, Fodstad O and Hovig E (1997) Ectopic expression of target genes may represent an inherent limitation of RT-PCR assays used for micrometastasis detection: studies on the epithelial glycoprotein gene EGP-2. *Int J Cancer* **72**: 191–196

Denis MG, Lipart C, Leborgne J, LeHur PA, Galmiche JP, Denis M, Ruud E, Truchaud A and Lustenberger P (1997) Detection of disseminated tumor cells in peripheral blood of colorectal cancer patients. *Int J Cancer* **74**: 540–544

Engel H, Kleespies C, Friedrich J, Breidenbach M, Kallenborn A, Schondorf T, Kolhagen H and Mallmann P (1999) Detection of circulating tumour cells in patients with breast or ovarian cancer by molecular cytogenetics. *Br J Cancer* **81**: 1165–1173

Friedl P, Noble PB, Walton PA, Laird DW, Chauvin PJ, Tabah RJ, Black M and Zanker KS (1995) Migration of coordinated cell clusters in mesenchymal and epithelial cancer explants in vitro. *Cancer Res* **55**: 4557–4560

Gyapay G, Morissette J, Vignal A, Dib C, Fizames C, Millasseau P, Marc S, Bernardi G, Lathrop M and Weissenbach J (1994) The 1993–94 Genethon human genetic linkage map [see comments]. *Nat Genet* **7**: 246–339

Hampl M, Hampl JA, Reiss G, Schackert G, Saeger HD and Schackert HK (1999) Loss of heterozygosity accumulation in primary breast carcinomas and additionally in corresponding distant metastases is associated with poor outcome. *Clin Cancer Res* **5**: 1417–1425

Harada Y, Katagiri T, Ito I, Akiyama F, Sakamoto G, Kasumi F, Nakamura Y and Emi M (1994) Genetic studies of 457 breast cancers. Clinicopathologic parameters compared with genetic alterations [see comments]. *Cancer* **74**: 2281–2286

Hardingham JE, Kotasek D, Farmer B, Butler RN, Mi JX, Sage RE and Dobrovic A (1993) Immunobead-PCR: a technique for the detection of circulating tumor cells using immunomagnetic beads and the polymerase chain reaction. *Cancer Res* **53**: 3455–3458

Hauge XY, Evans GA and Litt M (1991) Dinucleotide repeat polymorphism at the D11S528 locus. *Nucleic Acids Res* **19**: 1964

Heide I, Thiede C, Sonntag T, de Kant E, Neubauer A, Jonas S, Peter FJ, Neuhaus P, Hermann R, Huhn D and Rochlitz CF (1997) The status of p53 in the metastatic progression of colorectal cancer. *Eur J Cancer* **33**: 1314–1322

Huang Y, Boynton RF, Blount PL, Silverstein RJ, Yin J, Tong Y, McDaniel TK, Newkirk C, Resau JH, Sridhara R and et al (1992) Loss of heterozygosity involves multiple tumor suppressor genes in human esophageal cancers. *Cancer Res* **52**: 6525–6530

Kirchweger R, Zeillinger R, Schneeberger C, Speiser P, Louason G and Theillet C (1994) Patterns of allele losses suggest the existence of five distinct regions of LOH on chromosome 17 in breast cancer. *Int J Cancer* **56**: 193–199

Leitzel K, Lieu B, Curley E, Smith J, Chinchilli V, Rychlik W and Lipton A (1998) Detection of cancer cells in peripheral blood of breast cancer patients using reverse transcription-polymerase chain reaction for epidermal growth factor receptor. *Clin Cancer Res* **4**: 3037–3043

Liggett WH, Jr and Sidransky D (1998) Role of the p16 tumor suppressor gene in cancer. *J Clin Oncol* **16**: 1197–1206

Lizard Nacol S, Riedinger JM, Lizard G, Glasser AL, Coudray N, Chaplain G and Guerrin J (1997) Loss of heterozygosity at the TP53 gene: independent occurrence from genetic instability events in node-negative breast cancer. *Int J Cancer* **72**: 599–603

Lopez Guerrero JA, Bolufer Gilabert P, Sanz Alonso M, Barragan Gonzalez E, Palau Perez J, De la Rubia Comos J, Sempere Talens A and Bonanad Boix S (1997) Minimal illegitimate levels of cytokeratin K19 expression in mononucleated blood cells detected by a reverse transcription PCR method (RT-PCR). *Clin Chim Acta* **263**: 105–116

McGee TL, Yandell DW and Dryja TP (1989) Structure and partial genomic sequence of the human retinoblastoma susceptibility gene. *Gene* **80**: 119–128

Naume B, Borgen E, Nesland JM, Beiske K, Gilen E, Renolen A, Ravnas G, Qvist H, Karesen R and Kvalheim G (1998) Increased sensitivity for detection of micrometastases in bone-marrow/peripheral-blood stem-cell products from breast-cancer patients by negative immunomagnetic separation. *Int J Cancer* **78**: 556–560

Nawroz H, Koch W, Anker P, Stroun M and Sidransky D (1996) Microsatellite alterations in serum DNA of head and neck cancer patients. *Nat Med* **2**: 1035–1037

Nemoto T, Vana J, Bedwani RN, Baker HW, McGregor FH and Murphy GP (1980) Management and survival of female breast cancer: results of a national survey by the American College of Surgeons. *Cancer* **45**: 2917–2924

Offner S, Schmaus W, Witter K, Baretton GB, Schlimok G, Passlick B, Riethmuller G and Pantel K (1999) p53 gene mutations are not required for early dissemination of cancer cells. *Proc Natl Acad Sci USA* **96**: 6942–6946

Soria JC, Gauthier LR, Raymond E, Granotier C, Morat L, Armand JP, Boussin FD and Sabatier L (1999) Molecular detection of telomerase-positive circulating epithelial cells in metastatic breast cancer patients. *Clin Cancer Res* **5**: 971–975

van Leeuwen C, Tops C, Breukel C, van der Klift H, Fodde R and Khan PM (1991) CA repeat polymorphism at the D5S299 locus linked to adenomatous polyposis coli (APC). *Nucleic Acids Res* **19**: 5805

Villuendas R, Sanchez-Beato M, Martinez JC, Saez AI, Martinez-Delgado B, Garcia JF, Mateo MS, Sanchez-Verde L, Benitez J, Martinez P and Piris MA (1998) Loss of p16/INK4A protein expression in non-Hodgkin’s lymphomas is a frequent finding associated with tumor progression. *Am J Pathol* **153**: 887–897

Zippelius A, Kufer P, Honold G, Kollermann MW, Oberneder R, Schlimok G, Riethmuller G and Pantel K (1997) Limitations of reverse-transcriptase polymerase chain reaction analyses for detection of micrometastatic epithelial cancer cells in bone marrow. *J Clin Oncol* **15**: 2701–2708

*© 2000 Cancer Research Campaign*
