# Prognostic value of genomic alterations in minimal residual cancer cells purified from the blood of breast cancer patients

**DOI:** 10.1054/bjoc.2001.1753

**Published:** 2001-03


					Br J Cancer 83(12): 1664-1673
Prognostic value of genomic alterations in minimal residual
cancer cells purified from the blood of breast cancer patients
P Uciechowski, C Eder, B Bockman, B Suchy, G Driesel, S Jackel,
I Kusiak, H-J Grill and M Giesing
F Austrup should have appeared as first author of this paper. The
publishers wish to apologize for the deletion of his name.
ERRATUM
British Journal of Cancer (2001) 84(6), 874
(c) 2001 Cancer Research Campaign
doi: 10.1054/ bjoc.2001.1753, available online at http://www.idealibrary.com on
874

				

## 

**Correction to:** *British Journal of Cancer* (2000) **83**, 1664–1673. doi:10.1054/bjoc.2000.1501

